# The Nauheim Treatment of Heart Disease

**Published:** 1898-08-20

**Authors:** 


### THE NAUHEIM TREATMENT OF HEART DISEASE.

A careful review of recent literature on the Nauheim or Schott treatment of heart disease leads us to the conclusion that, so far at least as certain of its effects are concerned, the time for discussing its efficacy is past, however much difference of opinion there may be as to its mode of action or as to the meaning of some of the variations in physical signs which accompany and follow its application. What seems clear is that, although at Nauheim and at other places where the complete treatment can be carried out, results can be obtained which cannot be got in a bed-room, still there are certain details, certain parts of the treatment, which are available anywhere, and ought to become a part of the ordinary armamentorium of the general practitioner, unless, indeed, medical men are prepared to put down the whole thing as quackery, a thing which they have no right to do. We allude more especially to the resistance exercises. As is well known, although there are some whose main faith is in the baths, there are others who have had considerable success even without baths at all, and certainly many cases are, at the beginning of the treatment, so ill that baths are out of the question. In the discussion of the subject at the last meeting of the British Balneological and Climatological Society one speaker, a man of unimpeachable authority, told the following story: "He was about to enter a house when a medical man seized him and dragged him into his carriage. His friend described to him that since eight o'clock that morning he had been trying to keep a man alive. He was over 70, suffering from diabetes with mitral disease and failure of the heart's action; he had injected him with ether, strychnine, and digitalis, and could not get him to rally. He went to see the man; his pulse was so weak that he could not count it; he was panting and could scarcely speak. He . . . saw the patient's wife, to whom he explained his intention of giving him some of the Schott exercises. She protested and would not allow it. . . . Ultimately she consented, and he (the speaker), with the help of his friend, gave four of the finger and hand movements. At the end of the time he said he felt better, and as if a load had been removed from his chest. He asked his friend to repeat these methods two or three times a day. After the eighth day he called on the patient again, and was more than surprised to find him taking his wife in to dinner." It should be noted that this case was related to show the importance of dosage, but it does equally well for our purpose, as showing the sort of effect which may be obtained by the treatment, and as emphasising our statement that a treatment which can give such results should be at once mastered by the mass of the profession, and not be allowed to drift into the hands of masseurs and irresponsible outsiders. Such finger exercises are quite as well worth learning as are the actions of new drugs, and as to the dignity of the profession surely our position depends on our power of saving life and alleviating disease. The administration of medical gymnastics by the doctor himself is at least no more undignified than the administration of a douche or the epilation of a ringworm.

